# Peptide Receptor Radionuclide Therapy in Thyroid Cancer

**DOI:** 10.3389/fendo.2022.896287

**Published:** 2022-05-30

**Authors:** Sriram Gubbi, Christian A. Koch, Joanna Klubo-Gwiezdzinska

**Affiliations:** ^1^Metabolic Diseases Branch, National Institute of Diabetes and Digestive and Kidney Diseases, National Institutes of Health, Bethesda, MD, United States; ^2^Department of Medicine, Fox Chase Cancer Center, Philadelphia, PA, United States; ^3^Department of Medicine, The University of Tennessee Health Science Center, Memphis, TN, United States

**Keywords:** thyroid cancer, PRRT, DOTATATE, medullary thyroid cancer (MTC), somatostatin receptor

## Abstract

The treatment options that are currently available for management of metastatic, progressive radioactive iodine (RAI)-refractory differentiated thyroid cancers (DTCs), and medullary thyroid cancers (MTCs) are limited. While there are several systemic targeted therapies, such as tyrosine kinase inhibitors, that are being evaluated and implemented in the treatment of these cancers, such therapies are associated with serious, sometimes life-threatening, adverse events. Peptide receptor radionuclide therapy (PRRT) has the potential to be an effective and safe modality for treating patients with somatostatin receptor (SSTR)+ RAI-refractory DTCs and MTCs. MTCs and certain sub-types of RAI-refractory DTCs, such as Hürthle cell cancers which are less responsive to conventional modalities of treatment, have demonstrated a favorable response to treatment with PRRT. While the current literature offers hope for utilization of PRRT in thyroid cancer, several areas of this field remain to be investigated further, especially head-to-head comparisons with other systemic targeted therapies. In this review, we provide a comprehensive outlook on the current translational and clinical data on the use of various PRRTs, including diagnostic utility of somatostatin analogs, theranostic properties of PRRT, and the potential areas for future research.

## Introduction

Peptide receptor radionuclide therapy (PRRT) has emerged as one of the most pivotal modalities of treatment for neuroendocrine tumors (NETs). PRRT is a form of targeted therapy in which a radiolabeled peptide is used as a vector to deliver cytotoxic doses of radiation to those cancer cells which abundantly express receptors for that particular peptide (1). To date, several forms of radiolabeled peptides have been utilized for diagnostic purposes based on tumor receptor expression profiles, including somatostatin, cholecystokinin (CCK), neuropeptide-Y, gastrin, glucagon-like peptide-1, and others (2). However, the vast majority of PRRTs are radionuclides tagged to somatostatin analogs (SSAs), which target somatostatin receptors (SSTRs). While PRRT has been vastly utilized in the treatment of NETs, one of the actively investigated fields is the utility of PRRT in the treatment of thyroid cancer. In this review, we discuss the current standard-of-care for the management of various forms of thyroid cancer and then, discuss the utility of PRRT in oncology, along with the current evidence of SSA-based diagnostic imaging and PRRT in thyroid cancer. We also discuss the potential applicability of theranostics and future directions in the utility of PRRT as a crucial form of targeted therapy in thyroid cancer.

## Thyroid Cancers: The Current Standard of Care

The yearly incidence of thyroid cancer has nearly tripled from 4.9 per 100,000 individuals in 1975 to 14.3 per 100,000 individuals in 2009 (3). Differentiated thyroid cancer (DTC), comprised of papillary thyroid cancer (PTC), and follicular thyroid cancer (FTC) along with the Hürthle cell subtype of thyroid cancer (HTC), constitutes over 90% of all thyroid cancers (3). Medullary thyroid cancer (MTC) and anaplastic thyroid cancer (ATC) are much rarer but carry a higher mortality risk compared to DTC (4, 5).

The conventional treatment for DTC is thyroidectomy (hemi- or total thyroidectomy depending on the extent of the disease) with or without central and/or lateral lymph node dissection. Following surgery, radioactive iodine (RAI) therapy with iodine-131 (^131^I) is generally considered for those DTCs that have an intermediate-to-high risk for structural disease recurrence (3). For local recurrence and for distant metastases, repeat surgery and/or RAI therapy remains the preferred modality of treatment, and most RAI-avid DTCs have a favorable prognosis despite the presence of metastatic disease (3). However, up to 22% of DTCs may become RAI-refractory due to their inability to concentrate iodine into the tumor cells, and certain DTC subtypes such as tall-cell and diffuse sclerosing variants of PTC, insular variant of FTC, HTC, and poorly differentiated thyroid cancers (PDTCs) have a propensity to be RAI-refractory (3). In these circumstances, RAI is not effective, and diagnostic imaging is performed using 18-fluorodeoxyglucose positron emission tomography computed tomography (^18^FDG-PET/CT). In general, the more differentiated forms of DTC tend to retain iodine-concentrating capacity, are metabolically less active, more RAI-avid, and less ^18^FDG-PET-avid, while the more dedifferentiated forms are hypermetabolic, less RAI-avid, and more ^18^FDG-PET-avid (6). Local treatment options for RAI-refractory DTCs include external beam radiation, radiofrequency ablation or cryoablation. Molecular characterization of DTCs (7), and the advent of widespread molecular testing has allowed for identification of several potential targets for therapy. The tyrosine kinase inhibitors (TKIs) lenvatinib and sorafenib have been approved by the Food and Drug Administration (FDA) in the United States for the treatment of progressive, RAI-refractory metastatic DTC (8, 9). Other systemic therapies such as proto-oncogene serine/threonine-protein kinase (BRAF) inhibitors (dabrafenib), mammalian target of rapamycin (mTOR) inhibitors (everolimus), neurotrophic receptor tyrosine kinase (NTRK) inhibitors and immune checkpoint inhibitors (ICIs) are being evaluated in several clinical trials (10, 11). Recently, re-differentiation and restoration of iodine-concentrating capacity within RAI-refractory tumors by using mitogen-activated protein kinase kinase (MEK) inhibitors (trametinib and selumetinib) is being actively investigated as another promising modality of therapy (12, 13). Selumetinib has been granted an orphan drug designation by the FDA as an agent re-inducing RAI uptake in advanced DTC. Certain forms of DTCs such as HTCs, and PDTCs are rare and data on the utility of targeted therapy in these thyroid cancer subtypes is limited, although there is recent data emerging on long-term remission noted in PDTCs with the use of combination of lenvatinib (TKI) and pembrolizumab (ICI) (14, 15).

MTC is considered as a type of NET and is derived from the parafollicular C-cells (4). The prevalence of MTC is 1% – 2% in the United States (4). MTC can be sporadic or can manifest as a part of multiple endocrine neoplasia 2A and 2B. As the C-cells do not have the ability to concentrate iodine, MTCs are RAI-refractory, and the major functional imaging of utility is the ^18^FDG-PET/CT (4). The conventional treatment for MTC is thyroidectomy and lymph node dissection (4). Other treatments include external beam radiation for recurrent disease in the neck, surgical resection, or chemoembolization of hepatic metastases, and systemic therapy for progressive, metastatic MTC (4). Initially, two TKIs, vandetanib and cabozantinib, demonstrated efficacy in improving progression-free survival (PFS) in MTC and are now approved by the FDA for the treatment of progressive metastatic MTC (16, 17). Recently, two selective rearranged during transfection (RET) inhibitors, selpercatinib and pralsetinib have been approved for the treatment of progressive metastatic MTC, and these agents harbor better adverse event (AE) profiles (18, 19). Several other targeted therapies, including ICIs and carcinoembryonic antigen (CEA) vaccine are also being investigated for the treatment of this condition (11).

ATC is the most aggressive form of thyroid cancer, with a prevalence of 1.7% in the United States and a median prevalence of 3.6% worldwide (5). The management of ATC is more complicated due to the rapid progression and poor prognosis of the disease. For potential surgical resection of the primary tumor, several factors should be considered, including patient’s age, functional status, extent of the disease, status of airway, use of neoadjuvant or adjuvant therapy, whether the surgery is curative, palliative, or for prevention of complications such as obstruction of trachea and/or esophagus, and the goals of care of the patients, with timely involvement of palliative care (5). Neoadjuvant or adjuvant chemoradiation should be implemented as soon as possible after careful consideration of risks and benefits (5). Some of the available chemotherapy regimens include paclitaxel/carboplatin and docetaxel/doxorubicin, and their use is recommended in patients treated with definitive-intention radiation (5). In patients who have undergone R0 (negative microscopic and gross tumor margin) or R1 (negative gross tumor margin) surgical resection, or in patients with R2 resection (positive gross tumor margin/debulking procedure) with good performance status, intensity-modulated radiation with systemic therapy is recommended (5). However, the overall survival (OS), and disease-free survival is not different among R0, R1, and R2 statuses (20). In BRAF V600E-mutated ATCs, a combination of BRAF and MEK inhibitors can be utilized. The BRAF/MEK inhibitor combination of dabrafenib and trametinib is approved by the FDA for the treatment of BRAF V600E-mutated ATCs based on the substantial response rate observed in a phase II, open label trial (5, 21). Anaplastic lymphoma kinase (ALK) inhibitors, NTRK inhibitors, mTOR inhibitors, ICIs and TKIs (particularly the combination of lenvatinib and pembrolizumab) are some of the other targeted therapies with potential utility in ATC (5, 15, 22, 23).

Despite the availability of several local and systemic treatment options for thyroid cancers, metastatic/inoperable forms of RAI-refractory DTCs, HTCs, PDTCs, and MTCs continue to pose a challenge for optimal management. This is either due to suboptimal and variable response to treatment, or due to severe or unacceptable AEs or toxicities associated with several of these systemic therapies. Some of the common AEs associated with TKIs used for thyroid cancer include nausea, diarrhea, anorexia, fatigue, mucositis, hypertension, cardiac failure, rash, palmar-plantar erythrodysesthesia, skin pigmentary changes, alopecia, and aerodigestive fistula formation (24–27). The AE profile of selective RET inhibitors is similar to that of TKIs, with common AEs observed being neutropenia, hepatic transaminase elevation, constipation, diarrhea, hypertension, and rash (28). Some of the BRAF and MEK inhibitor-associated AEs include hypertension, reduced cardiac ejection fraction, cardiomyopathy, fever, diarrhea, rash, and ocular AEs such as central serous retinopathy (29–31). Therefore, there is a need for a systemic therapy that is not only efficacious, but also has lower number of AEs. PRRT can potentially serve as a systemic targeted therapy in thyroid cancer due to its better safety profile and high treatment efficacy demonstrated in several other forms of tumors (32–35).

## Somatostatin and SSTRs

Somatostatin, also known as somatotropin release inhibiting factor (SRIF) is a cyclic peptide hormone that has several complex endocrine, paracrine, and modulatory effects across different organ systems, including inhibition of secretion of various hormones and enzymes and cell proliferation, and also functions as a neurotransmitter and as an immunomodulator (36–39). Somatostatin was initially isolated from ovine hypothalamus as a peptide capable of inhibiting the release of growth hormone (40). Further studies have identified the presence of somatostatin in almost every tissue of the body (36, 41, 42). The two active forms of somatostatin are SRIF-14, which contains 14 amino acids and SRIF-28, which has 24 amino acids (43). Somatostatin is known to inhibit the secretion of not only growth hormone, but also of prolactin, thyroid stimulating hormone, adrenocorticotropic hormone, insulin, glucagon, and gastrointestinal hormones such as CCK, gastrin, ghrelin, secretin, and vasoactive intestinal polypeptide (38, 41, 44). Somatostatin also inhibits gastrointestinal exocrine function, including production of gastric, pancreatic, and intestinal digestive fluids and enzymes (38). Therefore, it has been recommended for routine use in patients undergoing pancreatic surgeries (45, 46).

SSTRs are a family of seven-subunit transmembrane G protein-coupled receptors, with five distinct subtypes being identified in humans, namely SSTR 1, 2, 3, 4, and 5 (37). The *SSTR* genes for each subtype are located on different chromosomes, and SSTR2 has two splice variants (SSTR2A and SSTR2B) (38). These SSTRs are vastly and variably expressed in different normal tissues as well as in various benign and malignant tumors in humans, but several forms of tumors seem to predominantly express SSTR2 and sometimes SSTR1, 3 or 5, while SSTR4 expression is rare (38, 39, 47, 48).

SRIF-14 and SRIF-28 bind to all five subtypes of SSTRs with nanomolar affinity (38). Furthermore, based on structural homology, the SSTRs are classified into two sub-groups, the SRIF1, which includes SSTRs 2, 3, and 5, and SRIF2, which includes SSTRs 1 and 4 (38). The synthetic SSAs such as octreotide and seglitide demonstrate binding affinity to SRIF1 receptors, with high affinity to SSTR2 and intermediate affinity to SSTR 1 and 5, while no such binding affinity is demonstrated towards SRIF2 receptors (49). Activation of all SSTR subtypes decreases adenylyl cyclase activity, and promotes variable effects on the activities of phospholipase C, mitogen-activated protein kinase, calcium and potassium channels, and phosphotyrosine phosphatase (38).

## SSTRs and SSAs in Oncology: The Advent of Theranostics

**Figure 1:**
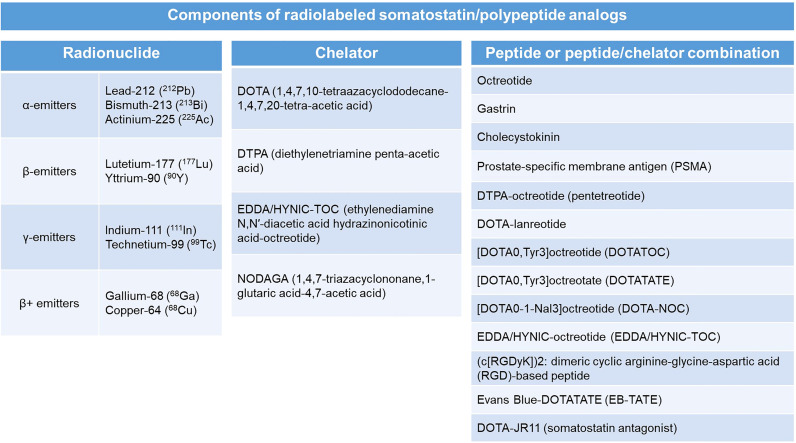
Components of a radiolabeled somatostatin analog with individual examples of each component.

Radiolabeled SSAs have emerged as some of the most widely used theranostic agents. Theranostics can be defined as the utilization of materials that can serve both as modalities of diagnostic imaging and as treatment (50). By combining diagnostics and therapeutics, theranostic agents harbor the advantage of sharing the same biodistribution and receptor selectivity that overcomes the potential disparity that may be encountered with using two different agents for imaging and treatment. The radionuclides tagged with the SSAs may serve a diagnostic or therapeutic purpose based on the type of the emitted radiation. Diagnostic imaging can be achieved by utilizing SSAs tagged with positron emitters such as gallium-68 (^68^Ga), copper-64 (^64^Cu) or fluorine-18 (^18^F) which can be imaged using positron (β+) emission tomography (PET), or with γ-emitters such as indium-111 (^111^In) or technetium-99 (^99^Tc) which can be imaged using single photon emission computed tomography (SPECT) (50). SSAs tagged with β-emitters such as lutetium-177 (^177^Lu) and yttrium-90 (^90^Y) or α-emitters such as lead-212 (^212^Pb), bismuth-213 (^213^Bi), and actinium-225 (^225^Ac) serve as therapeutic agents (PRRT) by destroying the targeted tumor cells (50). These radionuclides are chelated within various chelators such as DOTA (1,4,7,10-tetraazacyclododecane-1,4,7,20-tetra-acetic acid), DTPA (diethylenetriamine penta-acetic acid), or NODAGA (1,4,7-triazacyclononane,1-glutaric acid-4,7-acetic acid) and are ‘tagged’ to the SSAs. Earlier forms of PRRT comprised of conventional octreotide as the SSA. Later developments, such as modification of octreotide with insertion of tyrosine in the 3^rd^ position (Tyr^3^-OC or TOC), or replacing the c-terminal amino alcohol, threoninol, with threonine (TATE) improves the SSA’s binding affinity to SSTR2 subtype (51). Some of the common chelator-SSA combinations include [DOTA^0^,Tyr^3^]octreotate (DOTATATE), [DOTA^0^,Tyr^3^]octreotide (DOTATOC), and [DOTA^0^-1-NaI^3^]octreotide (DOTA-NOC) (35). The individual components of a radiolabeled SSA analog have been delineated in **Figure 1**. So far, ^177^Lu-SSAs, especially ^177^Lu-DOTATATE has been the most commonly utilized form of PRRT. Certain radiolabeled SSAs such as ^111^In-DTPA-octreotide (^111^In-pentetreotide) may not be as effective for PRRT when compared to ^177^Lu- or ^90^Y-based SSAs as the latter two radionuclides are β-emitters which are long-range electron emitters with better cytolytic potential and irradiation of the environment neighboring the tumor, while ^111^In is an Auger electron emitter which has short-range low-energy electron emission (51).

**Figure 2:**
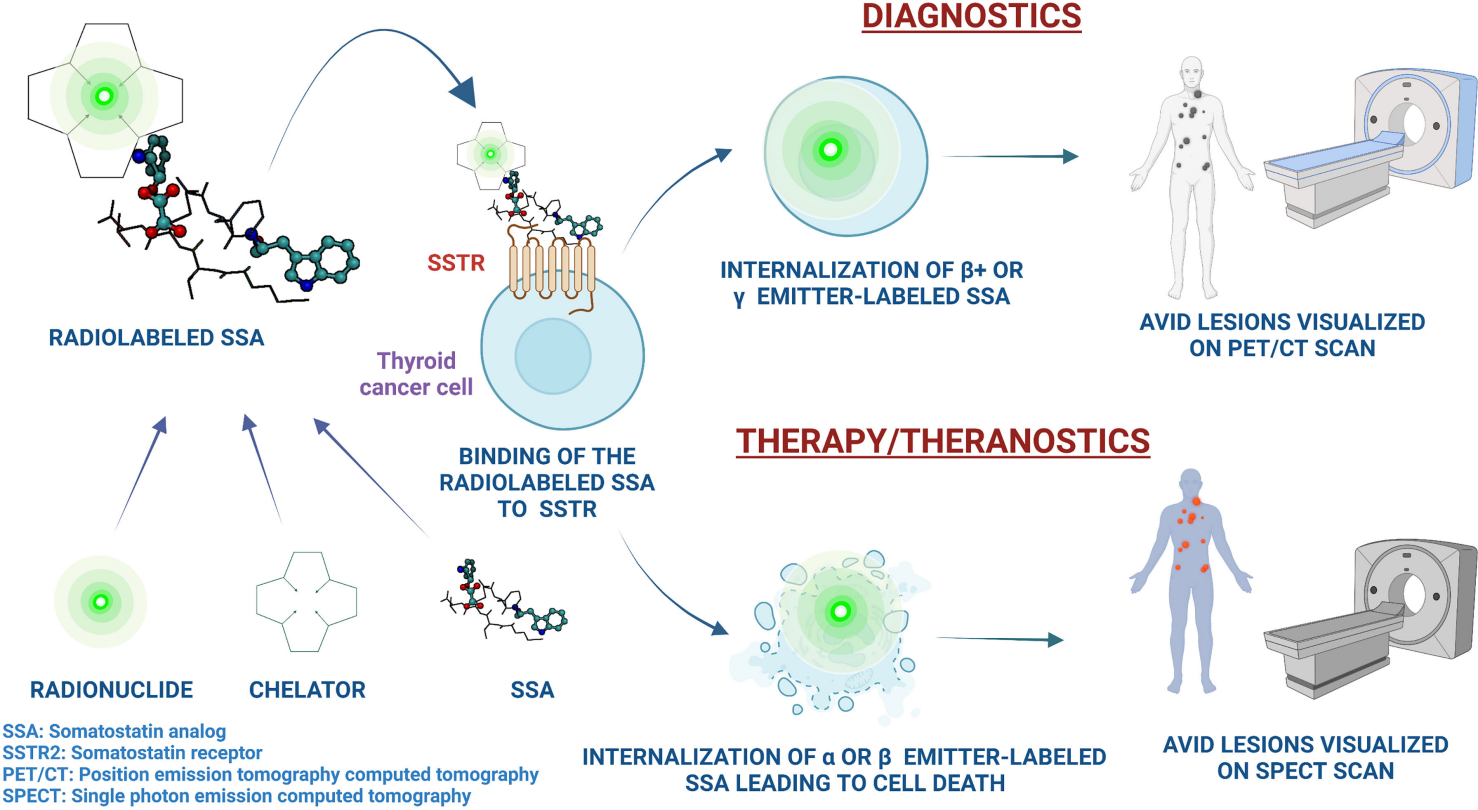
An illustration of the principles of utility of radiolabeled somatostatin analogs in the management of thyroid cancer, along with the mechanisms of action and the theranostic properties of peptide receptor radionuclide therapy. Image created on Biorender.com.

SSTRs are vastly overexpressed particularly in NETs; tumors that arise from neuroendocrine cells and/or neural crest cells (52). These cells are present in several organs, including the bronchi, pancreas, and the gastrointestinal tract. Based on the site of origin, NETs can be pancreatic NETs (PNETs), gastroenteropancreatic NETs (GEP-NETs), carcinoids, small cell lung cancer, or large cell neuroendocrine carcinoma (53). Other tumors of neuroendocrine origin include pheochromocytoma and paraganglioma (PPGL), MTC, neuroblastoma, meningioma, and Merkel cell carcinoma (53). In a landmark phase 3 clinical trial (NETTER-1), ^177^Lu-DOTATATE therapy in addition to long-acting repeatable (LAR) octreotide in patients with SSTR+ well-differentiated, metastatic midgut NETs resulted in substantially longer PFS compared to treatment with LAR octreotide alone (33). Based on the data from the NETTER-1 trial, the United States FDA approved ^177^Lu-DOTATATE in January 2018 for the treatment of SSTR+ GEP-NETs. The treatment was also approved in Europe based on the data from NETTER-1, as well as based on safety and efficacy data from the phase I/II ERASMUS study (34). In addition, PRRT has been effective in treating several other types of NETs, including PPGL (32, 54–60). The safety profile of PRRT is favorable and the treatment is generally well-tolerated. The most common AEs include nausea (65% all grades and 5% grades 3 and 4 in NETTER-1) and vomiting (53% all grades and 2% grades 3 and 4 in NETTER-1) which is mainly attributed to the renoprotective amino acid infusion that is concurrently administered with PRRT (61). This is managed with pre-infusion anti-emetic administration. Hematologic dysfunction, including transient myelosuppression, cytopenia (anemia: 81%, thrombocytopenia: 53%, neutropenia: 26% per NETTER-1 trial data, all grade 1 or 2), myelodysplastic syndrome (2.7% in NETTER-1, and 2% [median time to development: 28 months] in ERASMUS), and leukemia (0.5% [median time to development: 55 months] in ERASMUS) have been observed. Renal toxicity is of concern and <1% of patients in NETTER-1 trial developed renal failure, and this AE can be potentially prevented with amino acid infusion pre- and post-^177^Lu-DOTATATE administration. Other potential AEs include abdominal pain, constipation, diarrhea, hepatic dysfunction, hyperglycemia, electrolyte disturbances, and neurohormonal crises in cases of NETs producing biologically active products (33, 34). The principle of PRRT and the mechanism of action of radiolabeled SSAs has been illustrated in **Figure 2**.

## PRRT in Thyroid Cancers:

### SSTRs and SSAs in the Thyroid Gland and in Thyroid Cancer

*In vitro* studies from the 1990s have identified and characterized SSTR expression in normal thyroid tissue and in various thyroid cancer cell lines. One of the early studies showed that SSTRs 1, 3, and 5 were expressed in ATC cell lines while all SSTR subtypes were poorly expressed in PTC cell lines, and normal thyroid tissue predominantly expressed SSTR 3 and 5 (62). Administration of different types of SSAs to thyroid cancer cell lines, including those belonging to PTC, FTC, and ATC has demonstrated varying effects on cell growth depending on the cell line and the type of SSA (63). Similarly, MTC cell lines have also demonstrated avidity to somatostatin and to radiolabeled SSAs (64). SSTRs 1, 2, and 5 have been identified in tumor samples of patients with MTC, and differential effects on cell viability and on calcitonin and chromogranin A secretion has been demonstrated by the use of various receptor subtype agonists (65). Injection of ^111^In-pentetreotide and measurement of its activity in thyroid tumors and in peripheral blood in patients has revealed maximum tumor-to-blood ratio in MTCs, and while this ratio was lower in PTCs/HTCs compared to that of MTC, the ratio was still higher compared to normal thyroid and colloid goiters (66). Later studies have demonstrated SSTR2 and 5 expression in normal thyroid tissue and in DTCs (67–69). Klagge et al. identified expression of SSTRs 1, 2, 3, and 5 mRNA expression in normal thyroid and in various benign and malignant thyroid tumors, including PTC, FTC, and ATC (69). In this study, compared to normal thyroid tissue, expression of mRNA was significantly upregulated for SSTR2 in PTC, and for SSTR3 in PTC and ATC, while SSTR5 expression was non-significantly elevated in PTC and FTC. Our group also demonstrated a higher expression of SSTR2 in all types of thyroid cancers compared to normal thyroid based on immunohistochemical analysis of surgical tumor specimens obtained from patients with thyroid cancer (70). Furthermore, we identified *in vivo* uptake of ^68^Ga-DOTATATE in different thyroid cancers (70). While the maximum standardized uptake value (SUV_max_) was variable in PTCs and MTCs, the highest SUV_max_ values were observed with HTCs.

Normal thyroid gland tissue expresses SSTR2 and a physiologic uptake in the thyroid gland can be expected to be witnessed on ^68^Ga-DOTA SSA scans, with normal thyroid activity appearing equal to or lower than salivary gland or liver uptake (71, 72). However, in a retrospective analysis, atypical uptake patterns in the thyroid gland (diffusely increased homogeneous/heterogenous uptake, or focal increased uptake with a homogeneous normal thyroid uptake) were noted on diagnostic ^68^Ga-DOTATATE PET/CT scans performed in patients with NETs (72). In this study, of the 237 patients, 26 patients had atypical thyroid uptake, among whom 14 patients had focal uptake and 12 had diffuse uptake. Thyroid nodules were found in 10 out of 14 patients with focal uptake, and three out of these 10 patients were diagnosed with PTC. On the other hand, patients with diffuse uptake tended to have underlying hypothyroidism, thyroiditis, or non-toxic multinodular goiter. Similarly, uptake in normal thyroid, Hashimoto’s thyroiditis, autonomous thyroid nodule, and goiters have been observed on ^111^In-DTPA-octreotide (^111^In-pentetreotide) and ^68^Ga-DOTATOC scintigraphy (48, 73).

### Diagnostic Utility of Radiolabeled SSAs in Thyroid Cancer

In early studies, scintigraphic uptake of ^111^In-pentetreotide in the normal thyroid gland and in DTCs were identified, suggesting SSTR positivity in these tumors (74). In an early case series of 4 patients with metastatic thyroid cancers (2 RAI-refractory PTCs, and 2 ‘insular’ DTCs/PDTCs), ^111^In-pentetreotide scintigraphy revealed uptake in the tumors in both patients with insular variant, and in one PTC patient (75). Several other studies have evaluated ^111^In-labeled SSA scintigraphic uptake patterns in DTCs, including varying combinations of PTC, FTC, HTC, and PDTCs, with vast scintigraphy positivity rates ranging from 19% to 100% (51, 76–84). Binse et al. evaluated the diagnostic utility of ^68^Ga-DOTATOC in 15 consecutive patients with ^18^F-FDG-negative, RAI-refractory DTC with rising serum thyroglobulin (Tg) (85). ^68^Ga-DOTATOC uptake was demonstrated in 3/3 patients with PDTC, 1/1 patient with HTC, 1/5 patients with PTC, and 0/6 patients with FTC, and the serum Tg levels with ^68^Ga-DOTATOC-avid tumors showed a tendency to be higher. In another study by Kundu et al., ^68^Ga-DOTA-NOC-PET/CT was compared to ^18^F-FDG-PET/CT for diagnostic utility in 62 RAI-refractory DTC patients. Out of the 186 lesions identified on PET/CT, ^68^Ga-DOTA-NOC identified lesser number of lesions compared to ^18^F-FDG (65% vs. 90.3%; p <0.0001). However, ^68^Ga-DOTA-NOC influenced a change in management of disease in 34% patients as opposed to a change in management in 27% patients influenced by ^18^F-FDG. Using radio-isotopes that allow for more comprehensive detection of metastatic thyroid cancer is also of importance. In one study comparing intra-patient imaging differences between ^111^In-pentetreotide and ^99^Tc-ethylenediamine *N*,*N*′-diacetic acid hydrazinonicotinic acid-octreotide (^99^Tc-EDDA/HYNIC-TOC) in 11 thyroid cancer patients (8 PTC, 3 MTC), both modalities gave equivalent scintigraphic results, but ^99^Tc-EDDA/HYNIC-TOC managed to identify a solitary pulmonary metastatic lesion in 1 PTC patient, which was not visualized on the ^111^In-pentetreotide scan (86). Later studies have corroborated the diagnostic efficacy of ^99^Tc-EDDA/HYNIC-TOC in localizing metastatic disease in RAI-refractory DTCs, with particularly high sensitivities for detection of lymph node, bone, and lung metastases (87). There also lies a potential for utility of SSTR antagonists as pilot data has demonstrated superior tumor uptake noted on imaging on ^111^In-tagged SSTR antagonist when compared to ^111^In-pentetreotide (SSTR agonist) in NETs (88). Emerging data suggests that evaluation of tumor textural parameters rather than conventional parameters (such as SUV) on PET studies may be more predictive of survival outcomes in patients (89).

An early study utilizing ^111^In-octreotide scintigraphy demonstrated tumor uptake in 65% of patients with MTC, and scintigraphic uptake correlated with *in vitro* expression of SSTRs in these tumor samples (90). In a prospective study conducted on 30 patients with MTC, imaging with ^68^Ga-DOTATATE PET/CT demonstrated a superior sensitivity in identifying bone metastases compared to a conventional bone scan (91). Another study used ^111^In-pentetreotide and ^123^I/^131^I-metaiodobenzylguanidine (MIBG) for diagnostic imaging in 8 patients with metastatic MTC (92). Uptake was present in 5/8 patients with ^111^In-pentetreotide and in 4/8 patients with ^123^I/^131^I-MIBG imaging, and the number of metastatic lesions visualized on ^111^In-pentetreotide was higher than on ^123^I/^131^I-MIBG. In general, ^111^In-based SSA scintigraphy is positive in approximately 29% -77% of patients with MTC (92). In addition, CCK2 receptors are overexpressed in MTC, and scintigraphy DOTA-linked CCK agonists such as ^111^In-DOTA-CCK, ^111^In-DOTA-minigastrin 11, and ^99^Tc-demogastrin 2 for diagnostic imaging of MTC have yielded variable results (93). Other non-peptide-based radiolabeled compounds such as ^18^F-dihydroxyphenylalanine (DOPA) and ^18^F-dopamine have been evaluated for diagnostic imaging in MTC, and some results suggest a superior performance of ^18^F-DOPA in tumor detection compared to other functional imaging modalities, including those based on ^68^Ga-SSAs (94). However, head-to-head comparisons between ^18^F-DOPA and other radiolabeled SSAs (such as ^111^In-SSAs) or CCK-based imaging are yet to be assessed, and radiolabeled-SSAs are still of value in evaluating MTCs that demonstrate equivocal findings on ^18^F-DOPA or other functional imaging studies, and for selecting patients with SSTR+ tumors who can benefit from PRRT (94).

### Therapeutic Utility of Radiolabeled SSAs in Thyroid Cancer

A concise description of studies evaluating PRRT in thyroid cancer is provided in **Table 1**. Early studies demonstrated the efficacy of ^90^Y-DOTATOC and ^111^In-pentetreotide in patients with DTC (97, 99). Similarly, another early study demonstrated the efficacy of ^177^Lu-DOTATATE therapy in DTC (104). In this study, administration of 22.4 – 30.1 GBq of ^177^Lu-DOTATATE in 5 patients with ^111^In-pentetreotide-scintigraphy positive thyroid cancer demonstrated variable efficacy: SD in 1 PTC and 1 HTC, minor remission (MR; 25% - 50% tumor shrinkage) in 1 HTC, partial remission (PR; tumor shrinkage >50%) in 1 HTC, and PD in 1 FTC and 1 HTC patients. The 2 HTC patients with MR and PR demonstrated the highest ^177^Lu-DOTATATE uptake to pre-treatment ^111^In-pentetreotide uptake ratios. Several studies (sample size for DTC patients ranging from a single case to 25) were conducted in late 1990s to early 2000s to evaluate the efficacy of several forms of PRRTs consisting of ^111^In, ^90^Y- and ^177^Lu-labeled SSAs on a total of 62 DTC patients (18 PTC, 12 FTC, 7 HTC, and 25 unspecified thyroid cancers) either as a dedicated study for thyroid cancer or as a part of a larger study evaluating several other types of tumors (79, 82, 83, 86, 97–100, 103, 104). A total of 8% of population (5 patients, including 2 HTCs) achieved an objective tumor response while SD was noted in 42%, and time-to-progression (TTP) was inconsistently evaluated (51). To compare, in the phase 3 lenvatinib trial for RAI-refractory DTC (n=261 patients), after a median follow-up of 17.1 months, complete response (CR) was seen in 1.5%, PR in 63.2%, SD in 23%, and PD in 6.9% of the patients (8), and in the phase 3 sorafenib trial, the median TTP was 11.1 months (9). Later studies have also demonstrated the efficacy of ^90^Y and ^177^Lu-based PRRT in larger cohorts of DTC patients (106, 107, 110, 115) (**Table 1**), and the treatment was also associated with favorable safety profile. The most recent study by Roll et al. utilized ^177^Lu-DOTATATE (2 – 4 cycles in a span of 3 months; mean dose of 7 GBq) for treating 5 RAI-refractory SSTR2+ DTC patients with rising serum Tg levels (110). Based on post-therapy PET/CT and serum Tg values, 1 patient achieved PR, 3 patients had PD, while 1 patient had SD on imaging but had increasing serum Tg levels. These results suggest that a pre-treatment SSTR+ status of DTCs may not translate to effective treatment with ^177^Lu-DOTATATE, however, larger studies and optimization of SUV thresholds associated with response to therapy are needed to confirm the findings. Other ^177^Lu-based peptide or protein-tagged radionuclide therapies such as ^177^Lu-prostate-specific membrane antigen (PSMA) have been anecdotally utilized (116), although larger studies are required to establish the safety and efficacy of this particular PRRT.

**Table 1:** List of studies evaluating the efficacy of PRRT in thyroid cancer.

Study	Year	Number of patients	Number and type of thyroid cancer	Type of PRRT	Cumulative dose (GBq)	Duration of follow-up (months)	Outcomes*
1. Otte et al. (95)	1999	2	2 MTC	^90^Y-DOTATOC	1.6 – 2.9	Up to 2	2 SD (TTP = NA)
2. Caplin et al. (57)	2000	1	1 MTC	^111^In-octreotide	11.4	NA	NA
3. Paganelli et al. (96)	2001	3	3 MTC	^90^Y-DOTATOC	5.5 – 33.3	Up to 12	3 SD (TTP = NA)
4. Görges et al. (79)	2001	3	2 HTC 1 PTC	^90^Y-DOTATOC	1.7 – 9.6	16 – 27 (since last dose)	3 PD
5. Waldherr et al. (97)	2001	20	4 PTC 3 FTC 12 MTC 1 ATC	^90^Y-DOTATOC	1.7 – 14.8	Median: 15 (Range: 1 – 31)	7 SD (1 PTC, TTP = 8; 1 FTC, TTP = 8; 5 MTC, TTP = 3 – 14) 13 PD (3 PTC, 1 FTC, 7 MTC, 1 ATC)
6. Chinol et al. (98)	2002	17	2 PTC 15 MTC	^90^Y-DOTATOC	7.4 – 21.3	Median: 15 (Range: 2 – 47)	NA
7. Valkema et al. (99)	2002	11	4 PTC 1 FTC 6 MTC	111In-pentetreotide	5.8 – 87.3	Median: 14.4 (Range: 1.4 – 28.2)	Evaluated in 10 patients: 3 SD (1 PTC; 2 MTC TTP = NA) 7 PD (3 PTC, 1 FTC, 3 MTC)
8. Virgolini et al. (100)	2002	25	Unspecified	^90^Y-DOTA-lanreotide	0.9 – 7	Up to 36	3 MR (TTP = NA) 11 SD (TTP = NA) 11 PD
9. Christian et al. (82)	2003	1	1 HTC	^90^Y-DOTATOC	NA	NA	NA
10. Buscombe et al. (58)	2003	2	2 MTC	^111^In-pentetreotide	10.5 – 11.4	Up to 18	2 CR (TTP = NA)
11. Bodei et al. (101)	2003	8	8 MTC	^90^Y-DOTATOC	5.9 – 9.6	21 (4 – 26)	Evaluated in 7 patients: 1 CR (TTP = NA) 1 PR (TTP = NA) 3 SD (TTP = NA) 2 PD
12. Bodei et al. (102)	2004	21	21 MTC	^90^Y-DOTATOC	7.6 – 19.2	3 – 40	2 CR (TTP = NA) 12 SD (TTP = NA) 7 PD
13. Gabriel et al. (103)	2004	5	2 PTC 3 FTC	^90^Y-DOTATOC	5.6 – 7.6	At least 5 months	5 SD (TTP=minimum 5m)
14. Stokkel et al. (83)	2004	9	5 PTC 4 FTC	^111^In-pentetreotide	14.3 – 33.1	6 (since first dose)	4 SD (2 PTC, 2 FTC; TTP = NA) 5 PD (3 PTC, 2 FTC)
15. Gao et al. (92)	2004	1	1 MTC	^90^Y-DOTATOC	13.2	NA	1 SD (TTP = 6)
16. Pasieka et al. (56)	2004	1	1 MTC	^111^In-octreotide	11.8	9	1 PD
17. Teunissen et al. (104)	2005	5	1 PTC 1 FTC 3 HTC	^177^Lu-DOTATATE	22.4 – 30.1	0.5 – 63+	1 PR (1 HTC; TTP = 22m+) 1 MR (1 HTC; TTP = 43m) 2 SD (1 PTC; TTP = 18m and 1 HTC; TTP = 24+m) 1 PD (1 FTC; TTP = 4m)
18. Iten et al. (105)	2007	31	31 MTC	^90^Y-DOTATOC	1.7 – 29.6	Median: 12 (Range: 1 – 107)	Median OS: 16 (Range: 1 – 107)
19. Budiawan et al. (106)	2013	16	4 FTC 3 HTC 8 MTC 1 mixed FTC/MTC	^90^Y-DOTATOC ^177^Lu-DOTATATE	2.5 – 25 (^90^Y) 3.5 – 37.5 (^177^Lu)	NA	Evaluated in 12 patients: 1 PR (1 HTC, 1 MTC; TTP = NA) 1 SD (1 HTC, 3 MTC; TTP = NA) 5 PD (3 FTC, 1 MTC, 1 mixed FTC/MTC) Median PFS: 25 (IQR: 12 – 43)
20. Versari et al. (107)	2014	11	5 PTC 3 FTC 2 PDTC/insular 1 HTC	^90^Y-DOTATOC	4.3 – 18	NA	Evaluated in 10 patients: 2 PR (2 PTC, TTP = 7.5, 8) 4 SD (1 PTC, TTP = 3.5; 2 FTC, TTP = 7, 8; 1 HTC = 11.5) 4 PD
21. Vaisman et al. (108)	2015	7	7 MTC	^177^Lu-DOTATATE	29.6	8 – 12	3 PR (TTP = NA) 3 SD (TTP = NA) 1 PD
22. Lapa et al. (89)	2015	4	4 MTC	^177^Lu-DOTATATE	23.7 – 39	NA	4 PD
23. Salavati et al. (109)	2016	28	28 MTC	^90^Y-DOTATOC (n=NA) ^177^Lu-DOTATATE (n=NA)	NA	NA	5 PR (TTP = NA) 17 SD (TTP = NA) 6 PD Median OS: 72 in PR, 36 in SD, and 24 in PR
24. Roll et al. (110)	2018	5	1 PTC 3 FTC 1 HTC	^177^Lu-DOTATATE	14 - 28	NA	1 PR (1 FTC, TTP = NA) 2 SD (1 PTC, 1 HTC; TTP = NA) 2 PD (2 FTC)
25. Beukhof et al. (111)	2019	10	10 MTC	^177^Lu-DOTATATE	27.8 – 29.6	NA	4 SD 6 PD Median OS: 14 (Range: 5 – 144) Median PFS: 8 (Range: 4 – 144)
26. Parghane et al. (112)	2020	43	43 MTC	^177^Lu-DOTATATE	5.6 – 33.3	Median: 20 (Range: 8 – 78)	2 PR 25 SD 16 PD Median OS: 26 (95% CI: 16.6 – 35.3) Median PFS: 24 (95%.CI: 15.1 – 32.9)
27. Satapathy et al. (113)	2020	8	8 MTC	^177^Lu-DOTATATE + capecitabine	6.4 – 27.8	Median: 34 (Range: 14 – 69)	Evaluated in 7 patients: 6 SD (TTP = NA) 1 PD (TTP = NA)
28. Hayes et al. (114)	2021	21	21 MTC	4 ^90^Y-DOTATATE 1 ^90^Y-DOTATOC 5 ^177^Lu-DOTATATE 7 ^177^Lu-DOTATOC 4 ^90^Y-DOTATOC + ^177^Lu-DOTATOC	3.4 – 13.1 (^90^Y) 12.1 – 30.2 (^177^Lu)	Median: 65	Median OS: 63 (from 1^st^ cycle) Median TTF: 14

PRRT, peptide receptor radionuclide therapy; PTC, papillary thyroid cancer; FTC, follicular thyroid cancer; HTC, Hürthle cell thyroid cancer; MTC, medullary thyroid cancer; PDTC, poorly differentiated thyroid cancer; ATC, anaplastic thyroid cancer; CR, complete response; PR, partial response; SD, stable disease; PD, progressive disease; TTP, time-to-progression; OS, overall survival; PFS, progression-free survival; TTF, time-to-treatment failure; IQR, interquartile range; NA, not available.

*All outcomes data are provided in months.

Based on the available evidence in 2020, an executive committee comprised of experts from nuclear medicine, thyroid oncology, and head and neck surgery published the appropriateness of use of nuclear medicine studies in DTC (117). The committee deemed that diagnostic imaging with ^68^Ga-DOTATATE PET/CT should be considered in patients with RAI-refractory DTC in whom the serum Tg levels are rising and there is no identified source on conventional imaging, although they acknowledged the lack of sufficient evidence to correlate Tg levels or the rate of increase in Tg levels with the likelihood of detecting DTC on ^68^Ga-DOTATATE imaging. The committee also agreed that ^177^Lu-labeled SSAs may be useful in the treatment of RAI-refractory SSTR+ DTCs based on preliminary evidence, suggested by the observed objective response of 27% - 60% tumor burden reduction as per Response Evaluation Criteria in Solid Tumors (RECIST) criteria (107). Both ^68^Ga- and ^177^Lu-labeled SSAs were deemed inappropriate for use during pregnancy and lactation.

As MTC is considered as a form of neural crest tumor/NET, experience with PRRT in MTC is more robust as compared to DTC or ATC. PRRT in MTC has been evaluated by utilizing ^90^Y, ^177^Lu, and ^111^In radioisotopes, either tagged to SSAs or to CCK2 receptor agonists (56–58, 101, 109, 118, 119). Studies related to PRRT in MTC have been summarized in **Table 1**. Initial studies mainly utilized ^90^Y-DOTATOC as the mode of PRRT in small cohorts of MTC patients (95–97). In a retrospective analysis performed on 21 patients with SSTR+ metastatic MTC treated with ^90^Y-DOTATOC (7.5-19.2 GBq; 2 – 8 cycles), 2 (10%) patients achieved structural CR, 12 (57%) patients achieved stable structural disease, while seven (33%) patients did not respond to treatment, with a 3 – 40 months treatment duration (102). Biochemical (calcitonin and carcinoembryonic antigen [CEA]) CR was seen in 1 (5%) patient, PR in 5 (24%) patients, SD in 3 (14%) patients, and PD in 12 (57%) patients. In a phase II clinical trial, treatment with ^90^Y-DOTATOC in 30 adult patients with SSTR+ metastatic MTC was associated with response to therapy in 29% of the patients (105). This response was associated with longer survival from the time of diagnosis, and from the first ^90^Y-DOTATOC dose. Interestingly, response and survival were not associated with the visual grade of scintigraphic uptake (a 4-point scale of uptake pattern i. no uptake, ii. less than liver uptake, iii. similar to liver uptake, or iv. more than liver uptake) in the tumors, suggesting a potential for tumor response independent of uptake patterns on pre-therapy diagnostic scintigraphy. This observation is in contrast with the studies evaluating the association between the efficacy of ^177^Lu-DOTATATE in NETs, where SUV_max_ exceeding 13 – 15 by ^68^Ga-DOTATAE PET/CT was associated with better PFS (120, 121).

Later studies started evaluating the efficacy of ^177^Lu-based PRRT in MTC. In the study by Budiawan et al., both ^177^Lu-DOTATATE and ^90^Y-DOTATOC were used in a patient population with MTC as well as DTC (106). However, there was no discrete data on which patient received ^90^Y- vs. ^177^Lu-based PRRT. Among the 8 MTC patients included in the study, response to treatment was assessed in 5 patients after the last PRRT. PR was seen in 1 patient (20%), SD in 3 patients (60%), and PD in 1 patient (20%). Other studies also demonstrated the efficacy of ^177^Lu-DOTATATE therapy in small cohorts of MTC patients (108, 111) (**Table 1**), with one study noting improved quality of life among responders at 6 – 12 months post-therapy (108), and a higher uptake on the pre-PRRT diagnostic ^111^In-pentetreotide scans among patients with SD being noted in the other study (111).

More recent studies have analyzed the efficacy of PRRT on larger cohorts of patients. Parghane et al. evaluated the efficacy and toxicity of ^177^Lu-DOTATATE (average of 5.5 GBq; average of 3 cycles; range: 1 – 6 cycles) in 43 patients with SSTR+ metastatic MTC (112). The PFS was 24 months (95% CI: 15.1 – 32.9 months), and OS was 26 months (95% CI: 16.6 – 35.3 months) since the first dose of ^177^Lu-DOTATATE. A calcitonin doubling time of >24 months was associated with longer PFS, and OS compared to a doubling time of <6 months. Regarding biochemical response, CR was noted in 11%, PR in 30%, SD in 10%, and PD in 49% of the patients. The treatment was well-tolerated with grade 1 nausea and hemotoxicity and no nephrotoxicity or grade 3 or 4 toxicities were observed. To compare, the median PFS was 11.2 months in the cabozantinib trial among the patients in the cabozantinib treatment group (n = 219), and the predicted median PFS was 30.5 months in the vandetanib trial among patients receiving vandetanib (n = 231). In the NETTER-1 trial, patients receiving ^177^Lu-DOTATATE (n = 116) had an estimated PFS of 65%. Therefore, the PFS with PRRT in MTC seems to be comparable to the PFS observed with TKI therapy, although randomized controlled trials with standard-of-care therapies as control group are needed to appropriately compare these outcomes. Hayes et al. performed a retrospective analysis of data obtained from 71 patients with ^68^Ga-DOTATATE-avid metastatic MTCs to evaluate efficacy of PRRT (114). Among the 21 patients who received PRRT, the median OS was 63 months and the median time to treatment failure was 14 months, and a calcitonin doubling time of ≤24 months, age ≥60 years, and strong ^18^FDG-PET avidity were associated with poorer survival outcomes.

Another emerging strategy for enhancing PRRT efficacy is a combination therapy with a radiosensitizer such as capecitabine, which has been efficacious in treating GEP-NETs (59). In a retrospective observational study, data from 8 patients with inoperable/metastatic MTC treated with ^177^Lu-DOTATATE (median cumulative dose: 20.9 GBq; 1 – 4 cycles) along with capecitabine (1.25 g/m on days 1 – 14 of each cycle) were analyzed for treatment response and AEs (113). SD was noted in 7/8 (86%) patients, and PD was noted in 1 patient, and reduction in serum calcitonin was observed in 3/5 (60%) patients. There were no observed toxicities except for a grade 2 anemia.

To date, the utility of PRRT and SSA-based theranostics have been poorly studied in ATC. Some of the likely reasons include: highly dedifferentiated nature of the tumor which makes it unlikely to express SSTRs, the extremely rare prevalence of the tumor, and the rapid progression, possibly exceeding the rate of the rather slower therapeutic mechanism of radiation-induced apoptosis, and high morbidity and mortality associated with the disease. In a study that evaluated the treatment response of ^90^Y-DOTATOC in 20 thyroid cancer patients, 1 patient had progressive ATC, and ^90^Y-DOTATOC therapy failed to achieve an objective tumor response and the disease progressed during PRRT (97). Currently, imaging modalities based on targeting of galectin-3, a protein overexpressed in thyroid cancers, using radiolabeled monoclonal antibodies such as zirconium 89 (^89^Zr)-deferoxamine B-monoclonal antibody are being studied for ATC (122).

## Summary of Current Literature

Large-scale studies on PRRT in thyroid cancer are yet to commence. However, recently published systematic reviews and meta-analyses have allowed for evaluation of currently available data on the utility of PRRT in thyroid cancers. A meta-analysis by Lee et al. evaluated 11 published articles on DTC and/or MTC for efficacy and outcomes with various PRRTs (123). The objective response rate (ORR) in MTC was 8.5% (95% CI, 1.9% – 19.2%; i^2^ = 53.5%) and the disease control rate (DCR) was 60% (95% CI, 49.6%–69.8%; i^2^ = 28.7%), while for DTC, the ORR and DCR rates were 15.6% (95% CI, 7.80%–26.74%; i^2^ = 0%), and 53.9% (95% CI: 41.13%–66.39%; i^2^ = 0%), respectively. Proportion of serious/severe AEs (SAEs) was 2.8% (95% CI, 0.5%–8.3%) in MTC, and 2.82% (95% CI: 0.03%–17.61%) in DTC. In comparison, SAE frequency in other established systemic therapies for thyroid cancer have ranged from 0.4% to as high as 47% (**Table 2**), which further highlights the relatively better safety profile of PRRT. Also, ^177^Lu-based PRRT was associated with better ORR and DCR outcomes when compared to ^90^Y-based PRRT. In a systematic-review conducted by Maghsoomi et al. (124), on 41 studies related to PRRT in thyroid cancer (157 patients with RAI-refractory DTC and 220 patients with MTC), biochemical response was noted in 25.3% of DTC and 37.2% of MTC patients, and CR/PR were noted in 10.5% of DTC and 10.6% of MTC patients. In patients with advanced disease, 46 deaths out of 95 patients were noted in DTC and 63 deaths out of 144 patients were noted in MTC. While major AEs were noted with ^90^Y-based PRRT, the AEs with ^177^Lu- or ^111^In-based PRRTs were mild or transient. In comparison, in the NETTER-1 trial, the ORR was 18% (95% CI: 10% - 25%; 18/101 patients in the ^177^Lu-DOTATATE arm), while the frequency of SAEs was 9% in the ^177^Lu-DOTATATE arm (33). Among the TKIs, the ORRs have been between 12.2% - 64.8% for DTC, and 28% - 45% for MTC, and the SAE frequency is up to 30.3% in DTC and up to 15.9% in MTC (8, 9, 16, 17). With selective RET inhibitors, the ORR has been 60% - 73% for MTC and up to 79% - 89% in RET-fusion thyroid cancers, with SAEs up to 28% - 47% (18, 19). Although these are not head-to-head comparisons, PRRT for thyroid cancer seems to have comparable ORRs to PRRT in NETs, and while the ORRs are better with TKIs or with selective RET inhibitors, PRRT seems to have a better safety profile compared to these systemic therapies. A comparison of outcomes among the meta-analysis findings in PRRT and thyroid cancer, the various systemic therapies for thyroid cancer, and PRRT in NETs is provided in **Table 2**.

**Table 2:** A comparison of the outcomes of PRRT in thyroid cancer, other systemic therapies in thyroid cancer, and PRRT in NETs.

Study (year)	Type of study	Treatment	Tumor type (number of patients)	Duration of follow-up (months)	Outcomes
Lee et al. (2020) (123)	Meta-analysis	^177^Lu-SSA ^90^Y-SSA	DTC MTC (165)	NA	MTC: ORR: 8.5% DCR: 60% SAEs: 2.8% DTC: ORR: 15.6% DCR: 53.9% SAEs: 2.8%
Brose et al. (2014) – DECISION trial (9)	Phase 3 RCT, placebo-controlled	Sorafenib	DTC (207 in the treatment arm)	Median: 16.2	Median PFS: 10.8 months ORR: 12.2% DCR: 54.1% SAEs: 0.5% - 20.3%
Schlumberger et al. (2015) (8)	Phase 3 RCT, placebo-controlled	Lenvatinib	DTC (261 in the treatment arm)	Median: 17.1	Median PFS: 18.3 months ORR: 64.8% SAEs: 30.3%
Wells et al. (2012) (16)	Phase 3 RCT, placebo-controlled	Vandetanib	MTC (231 in the treatment arm)	Median: 24	Predicted median PFS: 30.5 months ORR: 45% DCR: 87% SAEs: 0.4% - 11%
Elisei et al. (2013) (17)	Phase 3 RCT, placebo-controlled	Cabozantinib	MTC (219 in the treatment arm)	Median: 13.9	Median PFS: 11.2 Median DOR: 14.6 ORR: 28% SAEs: 0.5% - 15.9%
Wirth et al. (2020) (18)	Phase 1/2 open-label	Selpercatinib	MTC RET-fusion-positive thyroid cancers (^a^55, ^b^88, ^c^19)	Median: 14.1^a^ Median: 7.8^b^ Median: 17.5^c^	ORR: 69% in patients previously treated with TKIs ORR: 73% in patients not previously treated with TKIs ORR: 79% in patients with RET-fusion-positive thyroid cancers SAEs: 1% - 28%
Subbiah et al. (2021) – ARROW study (19)	Phase 1/2 open-label	Pralsetinib	MTC RET-fusion-positive thyroid cancers (^a^61, ^b^23, ^c^11)	Median: 14.1^a^ Median: 10.8^b^ Median: 9.5^c^	ORR: 60% in patients previously treated with TKIs ORR: 71% in patients not previously treated with TKIs ORR: 89% in patients with RET-fusion-positive thyroid cancers SAEs: 1% - 47%
Strosberg et al. (2017) – NETTER-1 trial (33)	Phase 3 RCT, LAR octreotide alone as control group	^177^Lu-DOTATATE (with LAR octreotide)	Midgut NETs (116 in the PRRT arm)	34	Estimated PFS: 65% ORR: 18% SAEs: 9%

PRRT, peptide receptor radionuclide therapy; SSAs, somatostatin analogs; DTC, differentiated thyroid cancer; MTC, medullary thyroid cancer; NETs, neuroendocrine tumors; ORR, objective response rate; DCR, disease control rate; PFS, progression-free survival; DOR, duration of response; SAEs, serious/severe adverse events; TKIs, tyrosine kinase inhibitors; LAR, long-acting repeatable; NA, not available.

a Patients previously treated with TKIs.

b Patients not previously treated with TKIs.

c Patients with RET-fusion-positive thyroid cancers.

## Future Directions

Targeted therapy for thyroid cancer with PRRT is a continuously and rapidly evolving field, with several radiolabeled SSAs being investigated for their potential theranostic utility in various clinical trials for NETs. Nevertheless, the field of theranostics and PRRT in thyroid cancer is relatively young and has several areas that need further research. One such area includes further optimization of diagnostic radiolabeled ligand-based imaging for better visual delineation of thyroid tumors. As most PRRTs utilize SSAs that bind to SSTR2, treatment of thyroid cancers that express other types of SSTRs would not be feasible with most of the currently available PRRTs. Particularly DTCs may demonstrate varied SSTR subtype expressions as opposed to NETs, which commonly express SSTR2 (51). While several studies have demonstrated SSTR2 positivity in thyroid cancers, other studies have demonstrated a relative abundance of other SSTR subtypes, such as SSTR5, in these tumors (62, 66, 109, 125). These findings highlight the importance of developing PRRTs based on SSAs that could target SSTRs apart from the SSTR2 subtype. For instance, ^111^In-DOTA-NOC has increased affinity towards SSTR3 and 5, along with a high affinity to SSTR2, thus potentially allowing for further optimization of SSA-based tumor imaging (51, 126). In this manner, development of the corresponding ^177^Lu-DOTA-NOC or α-emitter-DOTA-NOC PRRTs may potentially be more efficacious in treating thyroid cancers.

A dosimetry-based approach to the diagnostic evaluation of thyroid cancers using radiolabeled SSAs allows for better identification of tumors that are likely to respond to the PRRT. The SUV_max_ values on 68Ga-DOTATOC imaging have been shown to correlate with SSTR2 mRNA expression in human tissues (67), and on the contrary, SUV_max_ correlates negatively with higher Ki-67 index (suggestive of more aggressive, undifferentiated tumor) among GEP-NETs (127). More dosimetric studies are required to further characterize the potential of thyroid cancers that can be targeted by PRRT. Individualized dosimetry-based approach focused on predicting the tumoricidal effects of certain tumor-absorbed dose thresholds has been proven effective in other types of cancers such as hepatocellular carcinoma (128). While β emitters are currently widely used as components of PRRT for NETs and thyroid cancer, α emitter-based SSAs also hold potential to serve as alternative forms of PRRT. As opposed to β emitters, which have a longer penetration range and a potential for damaging normal surrounding tissue, α emitters have a shorter penetration range leading to lesser normal tissue damage and have a higher energy and high linear energy transfer, leading to higher cytotoxicity (129, 130). More data on clinical utility of' α emitter-based PRRT should be available in the upcoming years. Another interesting area for further research is exploring methods to upregulate the expression of SSTRs on the cell surface. Preliminary *in vitro* and *in vivo* animal model experiments using epigenetic modifier drugs have resulted in successful upregulation of SSTR2 at transcriptional and functional levels (131). Further studies in humans are necessary to evaluate the safety and efficacy of SSTR-upregulator molecules. Literature on the association between PRRT and the molecular signatures of thyroid cancer (for instance, sporadic thyroid cancers versus thyroid cancers with germline pathogenic variants) is currently lacking and this area holds potential as another interesting avenue for future studies.

Apart from trying alternate radionuclides and chelators, modifications of the SSA or the peptide component of a radiolabeled SSA can also allow for improvement in the pharmacokinetics, and in turn potentially improve the diagnostic and therapeutic performance. Recently, Evans Blue (EB) analog has been conjugated with DOTATATE to develop DOTA-EB-TATE, which reversibly binds to the circulating albumin and improves the half-life of the molecule (132). ^90^Y-DOTA-EB-TATE has demonstrated better tumor response and survival rates compared to DOTATATE in mouse models (132). Our group also demonstrated that ^90^Y-DOTA-EB-TATE exhibits the highest tumor uptake in mouse xenograft models, irrespective of the type of thyroid cancer cell line xenograft, when compared to ^68^Ga-DOTATATE and ^68^Ga-DOTA-JR11 (70). Moreover, we also demonstrated superior tumor volume reduction and disease-specific survival in the SSTR2+ thyroid cancer xenograft mouse models with the use of ^177^Lu-DOTA-EB-TATE when compared to ^177^Lu-DOTA-TATE and^177^Lu-DOTA-JR11 (70). Therefore, EB-TATE-based SSA imaging and PRRT holds potential to improve theranostics in thyroid cancer.

Currently, clinical trials on PRRT or SSA-based imaging in thyroid cancers are sparse. For instance, one trial (NCT04106843) had to be withdrawn due to lack of registration of participants. There is one study (NCT04927416) that is currently recruiting patients for further characterization of metastatic RAI-refractory DTCs, HTCs, and MTCs using ^68^Ga-DOTATATE PET/CT. Another randomized cross-over study utilizing ^177^Lu-PP-F11N, a CCK2 receptor analog with or without sacubitril for treatment of metastatic MTC (NCT03647657) has been completed, and the results are awaited, while another combined pilot and phase I clinical trial (NCT02088645) is currently recruiting advanced MTC patients for theranostics with ^177^Lu-PP-F11N.

In conclusion, PRRT is a rapidly evolving field, and holds great potential as a theranostic tool in the management of metastatic RAI-refractory thyroid cancers. Current literature has provided substantial evidence for the efficacy and the safety profile of PRRT. Certain forms of thyroid cancers, such as HTCs and MTCs could be potentially treated better with PRRT as opposed to other forms of systemic therapies. The efficacy of PRRT may also depend on the tumor characteristics observed on the diagnostic radiolabeled-SSA imaging, and the molecular landscape of the various thyroid cancers. Large-scale, randomized controlled trials are necessary to further delineate the functional and molecular characteristics of the tumors that do and do not respond to PRRT and to perform head-to-head comparisons with other commonly used systemic therapies for the management of RAI-refractory thyroid cancers.

## Author Contributions

SG prepared the manuscript. CK reviewed the manuscript and provided suggestions to improve the manuscript. JK-G conceptualized the manuscript, reviewed the manuscript, and provided suggestions to improve the manuscript. All authors were involved in the revision of the manuscript. All authors contributed to the article and approved the submitted version.

## Funding

This work was funded by the intramural program of the National Institute of Diabetes and Digestive and Kidney Diseases of the National Institutes of Health.

## Conflict of Interest

The authors declare that the research was conducted in the absence of any commercial or financial relationships that could be construed as a potential conflict of interest.

## Publisher’s Note

All claims expressed in this article are solely those of the authors and do not necessarily represent those of their affiliated organizations, or those of the publisher, the editors and the reviewers. Any product that may be evaluated in this article, or claim that may be made by its manufacturer, is not guaranteed or endorsed by the publisher.

## Abbreviations

PRRT, peptide receptor radionuclide therapy; SSA, somatostatin analog; DTC, differentiated thyroid cancer; PTC, papillary thyroid cancer; FTC, follicular thyroid cancer; HTC, Hürthle cell thyroid cancer; MTC, medullary thyroid cancer; PDTC, poorly differentiated thyroid cancer; ATC, anaplastic thyroid cancer; NET, neuroendocrine tumor; RAI, radioactive iodine; ORR, objective response rate; DCR, disease control rate; OS, overall survival; PFS, progression-free survival; TTP,time-to-progression; TTF, time-to-treatment failure; DOR, duration of response; IQ, interquartile range; NA, not available; SAE, serious/severe adverse event; FDA, Food and Drug Administration; TKI, tyrosine kinase inhibitor; MEK, mitogen-activated protein kinase kinase; RET, rearranged during transfection; mTOR, mammalian target of rapamycin; NTRK, neurotrophic receptor tyrosine kinase; ICI, immune checkpoint inhibitor; ALK, Anaplastic lymphoma kinase; SRIF, somatotropin release inhibiting factor; LAR, long-acting repeatable; CT, computed tomography; MRI, magnetic resonance imaging; PET/CT, positron emission tomography computed tomography; SPECT, single photon emission computed tomography; FDG, fluorodeoxyglucose; DOPA, dihydroxyphenylalanine; MIBG, metaiodobenzylguanidine; DOTATOC, 1,4,7,10-tetraazacyclododecane-1,4,7,20-tetra-acetic acid-octreotide; DOTATATE, 1,4,7,10-tetraazacyclododecane-1,4,7,20-tetra-acetic acid-octreotate; DTPA, diethylenetriamine penta-acetic acid; DOTA-NOC, [DOTA0-1-NaI3]octreotide; NODAGA, 1,4,7-triazacyclononane,1-glutaric acid-4,7-acetic acid; (c[RGDyK])2, dimeric cyclic arginine-glycine-aspartic acid (RGD)-based peptide; EDDA/HYNIC-TOC, ethylenediamine N,N′-diacetic acid hydrazinonicotinic acid-octreotide; EB-TATE, Evans blue-DOTATATE; PSMA, Prostate-specific membrane antigen; CCK, cholecystokinin.
